# Proposal: Apparatus for Sensing the Effect of Surface Roughness on the Surface Resistance of Metals

**DOI:** 10.3390/s23010139

**Published:** 2022-12-23

**Authors:** Kostiantyn Torokhtii, Andrea Alimenti, Pablo Vidal García, Nicola Pompeo, Enrico Silva

**Affiliations:** Department of Industrial, Electronic and Mechanical Engineering, Roma Tre University, Via Vito Volterra 62, 00146 Roma, Italy

**Keywords:** surface roughness, dielectric resonator, nondestructive measurements, microwave surface resistance

## Abstract

The root mean square surface roughness $R_{q}$ of metals is detrimental in several microwave applications. $R_{q}$ characterization methods are thus largely used and of great interest. In this work, a new dielectric loaded resonator (DR) design is proposed to evaluate the surface resistance variations of samples with different $R_{q}$. The new design is thought to make the measurement accuracy, usually strongly affected by the measurement repeatability, suitable for this study. We analyze the measurement method’s sensitivity and accuracy in order to assess the possibility of using this new DR design for highly accurate surface resistance measurements sensitive to $R_{q}$ variations.

## 1. Introduction

One important characteristic of metallic surfaces is their roughness. In many application fields, surface roughness measurements can be a critical issue both for the maintenance of the performance of devices and during the design of particularly sensitive systems. To mention a few examples, the performance of electrochemical electrodes, which is in fact directly dependent on their roughness coefficient [1,2,3], the quality of the contact between two conductors, or the failing of copper wires under long-term mechanical stress [4,5]. Particular attention has been devoted to treatments to reduce the roughness of the conducting surface of accelerating cavities to minimize the residual resistance at radio and microwave frequencies [6,7]. This widespread interest in the effects of surface roughness on various physical properties stimulated the development of several methods to evaluate the surface roughness, either in contact or contactless setups [8]. For the case of a sensitive conducting surface, the use of non-destructive or even contactless methods is critical. Contactless methods are typically based on optical interferometry with resolution around 0.1μm [9,10]. On the other hand, it is not necessary to perform a multidimensional interferometric scanning measurement for applications where only roughness level indications are needed, or where the important information resides in the effect of the surface roughness and not in the absolute evaluation of the surface roughness itself. The case of interest in this paper is the evaluation of the effect of surface roughness on the microwave surface resistance of metals.

At high frequencies, the material property usually used to describe the conduction performance of good conductors is the surface resistance *R*. From classical electrodynamics it can be shown that, at microwaves (μw) and in thick conductors, $R=\sqrt{\omega \mu_{0}\rho /2}$ [11], where ρ is the material’s dc resistivity, ω is the angular frequency of the impinging electromagnetic (e.m.) field, and $\mu_{0}$ is the vacuum permittivity.

The use of materials with different *R* with respect to that used in the design of μw devices can lead to the production of components (e.g., larger filter bandwidth, signal distortion) that are out of specifications, since the μw power losses are proportional to *R* [11,12,13,14]. Thus, *R* is a particularly relevant design parameter. However, it is well-known that the microwave measured *R* is often far from the expected one obtained by dc measurements [15,16]. Understanding why the microwave measured *R* was normally unexpectedly large was a longstanding problem [15,16], and is still of great relevance [17,18]. It is now known that, when the conductor surface is scratched and rough, the measured *R* (and thus the high-frequency conduction losses) increases. In particular, it was observed that *R* rises when the root mean square (rms) of the vertical deviations of the roughness profile from the mean level, $R_{q}$ [19], approaches the e.m. skin depth $\delta =\sqrt{2\rho /(\omega \mu_{0})}$. *R* saturates when $R_{q}\gg \delta$ [20].

Despite the necessity to take into account metallic surfaces, there are no non-destructive experimental methods at GHz frequencies for the determination of surface roughness. Many studies on the modeling of different roughness profiles [21,22,23,24,25], or “simple” empirical formulae [20,26,27], are present in the literature. Since the results of these models are sometimes different and their application limits are not always clear [28], direct measurements of the function $R(R_{q})$ are of interest.

From a metrological point of view, an accurate evaluation of these models can be used to find the absolute *R* of conductive samples once their ρ and $R_{q}$ are known, to be used as standard references for the calibration of measurement devices (e.g., resonators), as in [29]. A different approach is the development of a highly accurate μw measurement device able to evaluate the *R* variations due to $R_{q}$. This approach can be useful in μw research laboratories where μw measurement devices are already present, and where the main concern is the evaluation of *R* in samples of known $R_{q}$.

In this work, we propose the use of a dielectric loaded resonator (DR) for the evaluation of the effects of $R_{q}$ on *R* of conductive samples. The use of a microwave technique to measure *R* is a straightforward way to assess the effect of $R_{q}$ on a property of direct interest. Among possible microwave methods, resonant methods are the most sensitive. In fact, resonant techniques are well-known for their high sensitivity and non-destructive nature, but they are equally known for their characteristic poor measurement repeatability [11]. Thus, to make these devices suitable for the aim of this work, a new design is useful to overcome their characteristic limits. We propose and realize a resonator where the sensitivity and accuracy are sufficient to measure typical changes in *R* as averaged over the sample under study, typical of the changes due to $R_{q}$.

The work is organized as follows. In Section 2 the measurement method is introduced, and in Section 3 the measurement setup is shown and its accuracy limits analyzed.

## 2. Measurement Method

In this work we propose the use of a DR for the evaluation of the $R(R_{q})$ of planar metal samples. Since the quality factor is defined as $Q=\omega_{0}W/P$ [11], with *P* the sum of all the losses inside the resonator, *W* the energy stored at the resonance and $\omega_{0}$ the resonance angular frequency, and considered that the ohmic losses of a metal surface $P_{\Omega }$ are proportional to *R*, then $Q^{-1}\propto R$. Thus, the DR *Q* can be used for *R* measurements using the following relation [11]:

(1) $$Q^{-1}=\sum_{i}\frac{R_{i}}{G_{i}}+l_{v}\;,$$

where $G_{i}$ is the geometrical factor of the *i*-th metal surface with surface resistance $R_{i}$ and $l_{v}$ is the overall volume losses contribution to *Q*. In order to obtain the *R* of the sample under investigation from (1), it is necessary to evaluate the contribution given by all the components of the resonator.

Since the aim of this work is the experimental evaluation of $R(R_{q})$, it is not possible to calibrate the resonator by computing the values of *R* of all the surfaces starting from dc ρ measurements as in [29]. For this reason, new calibration procedures or a perturbative approach are necessary [11]. In particular, once a reference sample with $R=R_{ref}$ is chosen, the difference $\Delta R=R_{s}-R_{ref}=G_{s}\left(Q_{s}^{-1}-Q_{ref}^{-1}\right)$ is obtained from (1) if the field configuration is not changed between the measurements. We indicate with the subscript “*s*” the quantities related to the sample under test; thus, $Q_{s}$ is the quality factor measured when the sample is loaded into the DR and $Q_{ref}$ when the reference is used. If the reference is chosen to be the sample with the lowest $R_{q}$ possible, then the variation of the surface resistance with respect to the variation of the surface roughness $\Delta R_{s}\left(\Delta R_{q}\right)$ is accessible.

## 3. Measurement System: Set-Up and Performance Analysis

In this section we present the measurement system designed for this application and we analyze its performances in order to assess its suitability for $R_{q}$ evaluation.

### 3.1. Measurement Set-Up

We designed a Hakki–Coleman dielectric loaded resonator working with the TE_011_ e.m. mode at ∼12.9 GHz. This geometry was chosen for the typically high sensitivity of these resonators [11], which is also demonstrated by their wide use in the characterization of low-loss conductive materials such as superconductors [11,30,31,32]. In our case, one base of the resonator is composed by a thin metal plate (here called a mask) with a circular hole in its centre. The mask allows for different-shaped samples to be measured without the need to disassemble the whole DR structure. This was a fundamental requirement of the design of the DR useful to reach the low measurement uncertainty needed for this application. In fact, it is well-known that mounting repeatability is a critical aspect in microwave resonant fixtures. Thus, the possibility of changing the sample under investigation without unmounting the whole structure can strongly improve the measurement precision.

**Figure 1:**
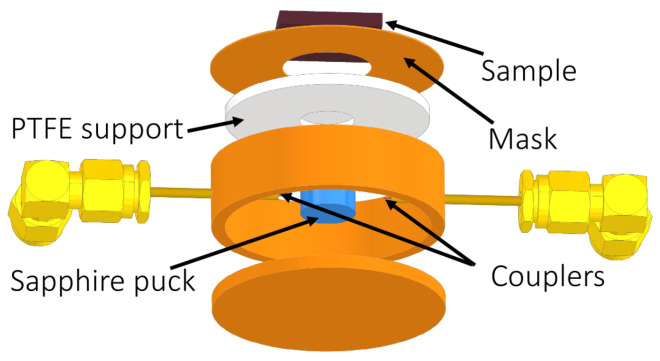
Structure of the proposed dielectric loaded resonator.

Figure 1 shows a sketch of the designed DR. The used dielectric crystal is a sapphire single-crystal cylinder (8.00 ± 0.01) mm in diameter and (5.00 ± 0.01) mm in height, fixed in the center of the cylindrical cavity by a PTFE support. The (0.20 ± 0.01) mm–thin brass mask hole with a diameter (13.00 ± 0.01) mm is designed to be compatible with the size of the samples under study (the hole diameter can be adapted to the size of the sample). The masks allow samples of different shapes to be measured without the need to disassemble the entire DR structure: the sample contribution to the response of the DR is determined by the hole diameter.

We use an Anritsu 37269D vector network analyzer set to acquire 1601 data points for each frequency sweep. The IF filter is set at 10 kHz and the frequency span is chosen to be 5 times larger than the resonance curve full width at half maximum [33].

The unloaded quality factor *Q* is measured from the transmission scattering parameter $S_{21}$ through the fit of the resonance curve with the modified Lorentzian model described in [34]. The resonator coupling is held sufficiently low (<0.005) to be negligible for the *Q* evaluation.

As expected, the mounting sensitivity, although close in magnitude to the fitting uncertainties thanks to the proposed design, remains the main limitation to the measurement precision.

### 3.2. Uncertainty Analysis

With the standard uncertainties propagation procedure, from (1) we obtain:

(2) $$\begin{array}{c} u^{2}\left(R_{1}\right)={\left(\frac{\partial R_{1}}{\partial Q}u\left(Q\right)\right)}^{2}+\sum_{i=1}^{N}{\left(\frac{\partial R_{1}}{\partial G_{i}}u\left(G_{i}\right)\right)}^{2}+\\ +\sum_{i=2}^{N}{\left(\frac{\partial R_{1}}{\partial R_{i}}u\left(R_{i}\right)\right)}^{2}+{\left(\frac{\partial R_{1}}{\partial l_{v}}u\left(l_{v}\right)\right)}^{2}\;, \end{array}$$

where the sample surface resistance $R_{s}=R_{1}$. The first term is a random contribution related to *Q* measurement precision while the others introduce a systematic contribution related to the resonator calibration.

**Figure 2:**
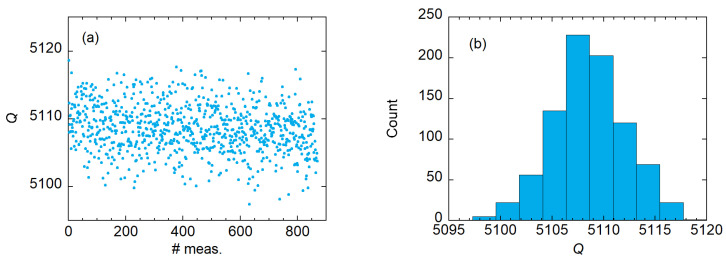
A total of 800 repeated *Q*-factor measurements were made with the resonator kept at fixed room temperature and without unmounting the sample. Measurement repetition and measurement histogram are shown in panels (**a**) and (**b**), respectively.

**Figure 3:**
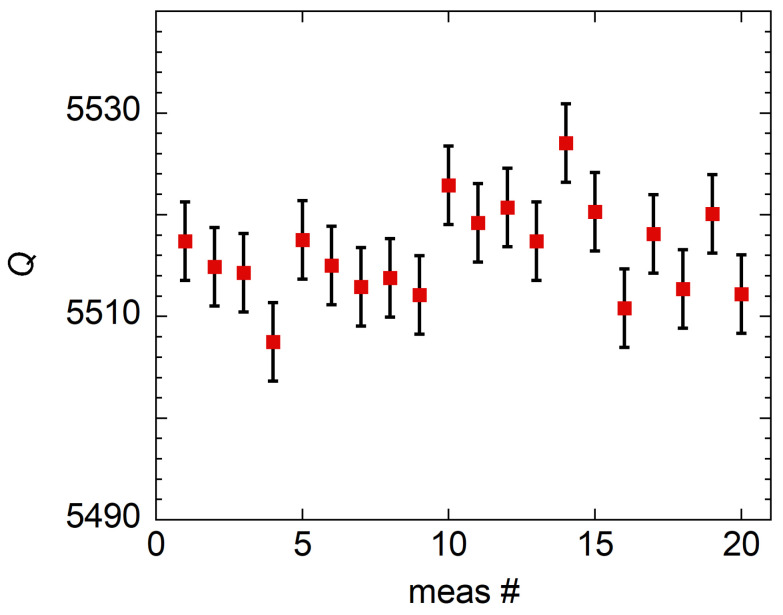
Mounting repeatability of the *Q*-factor measurement. The uncertainty bars represent the fitting uncertainties $u\left(Q\right)/Q∼10^{-4}$.

*Q* is obtained by fitting the complex transmission scattering *S*-parameters with the 6-parameters modified Lorentzian model [34,35]. For the evaluation of the quality factor uncertainty u(Q), several contributions must be considered:

- $S_{21}$ noise effect on fit precision/accuracy: the uncertainty $u{\left(Q\right)}_{noise}$ given by the electrical noise on the measured *S*-parameters is evaluated by the fit residuals variance [34] yielding $u{\left(Q\right)}_{noise}/Q∼$ 0.07%. The $u{\left(Q\right)}_{noise}$ amplitude is evaluated without the transmission line calibration applied in order to separate the two contributions. The same $u{\left(Q\right)}_{noise}$ is obtained by acquiring several resonance curves all in the same environmental condition and evaluating the *Q* standard deviation. The stability of the resonator was evaluated by repeating the *Q* measurements in a 3.5 h time period while keeping the room temperature as stable as possible. The 800 repeated measurements are shown in Figure 2. From these repetitions, we obtained $\bar{Q}\approx 5108.6$ and an experimental standard deviation of the sample of s(Q)≈3.5. Hence, $s\left(Q\right)/\bar{Q}\approx 7\times 10^{-4}$.
- Calibration: the resonance curves are acquired, then a full 12-term standard Short-Open-Load-Through calibration procedure is performed. With the calibration applied it is possible to evaluate the $u(S_{21})$. To evaluate how $u(S_{21})$ propagates to $u{\left(Q\right)}_{cal}$ we conducted a Monte Carlo simulation with 1000 noiseless resonance curves randomly varying for each iteration 1601 $S_{21}$ points in the uncertainty limits given in the vector network analyzer datasheet. In particular, in our measurement conditions, $u(|S_{12}|)<$ 0.2 dB and u(S12)<2∘. For the simulation we considered uniformly distributed $S_{12}$ points between 0 and the declared upper uncertainty limit. The simulated curves are then fitted and the standard deviation of the measured *Q* allows us to assess $u{\left(Q\right)}_{cal}/u\left(Q\right)∼0.07\%$.
- Measurement repeatability: we test the *Q* repeatability by performing 20 measurements, in each one mounting and disassembling the metal sample on the resonator. Thanks to the newly designed resonator, a low standard deviation of $s\left(Q\right)/\bar{Q}∼$ 0.11% is obtained. The *Q*-factor measurement repetitions are shown in Figure 3.

The effects of the different uncertainty sources on *Q* are then added in quadrature and the overall u(Q)/Q< 0.2%, which is mainly dominated by the measurement repeatability.

The u(Q) so evaluated can be compared with values in the literature to assess the goodness of the designed DR. However, careful evaluations of the metrological characteristics of microwave measurement fixtures are often overlooked and, in the few cases where uncertainties are declared, information on the evaluation procedure is not reported. For this reason, a performance comparison is not a trivial task. In [36], a relative uncertainty of 1% is declared for a dielectric-loaded resonator for cryogenic measurements, but the mounting repeatability is not reported. In [37] the authors assess that the measurement repeatability is typically on the order of a “few” percent for their resonating fixture. Finally, the international standard [38], based on a dielectric-loaded resonator for the surface impedance measurements of superconductors, reports a 4% relative standard uncertainty on *Q* (without considering the mounting repeatability). We can conclude that the reported values of the *Q*-factor measurement uncertainty (even when not including repeatability) are always about one order of magnitude greater than the value here obtained.

In the small perturbation limit, the geometrical factors $G_{i}$ do not depend on the metal samples’ properties, but instead only on the e.m. field configuration [11]. Thus, $G_{i}$ can be reliably evaluated through e.m. simulations. The uncertainties $u(G_{i})$ are evaluated with a Monte Carlo simulation on the e.m. simulator, randomly varying the physical dimensions of the DR in their variability space. The obtained uncertainty is $u(G_{i})∼$ 1%.

### 3.3. Differential R Measurement—End Wall Perturbation

Generally, DR calibration could be possible with standard metal samples and a dielectric sample of known properties. Nevertheless, it is not possible to reliably use *R* computed from dc measurements for the problems recalled in the previous section. For this reason a perturbative approach is suggested, and only the surface resistance variation $\Delta R_{s}=R_{s,1}-R_{s,2}$ between two samples (subscripts “s,1” and “s,2”) is evaluated. In fact, when the differences $\Delta R_{s}$ are considered, the systematic contributions are mainly canceled and no calibrations are needed. Thus, $\Delta R_{s}$ is obtained, performing two different measurements $Q_{s,1}$ and $Q_{s,2}$:

(3) $$\Delta R_{s}=G_{s}\left(Q_{s,1}^{-1}-Q_{s,2}^{-1}\right)\;.$$

Thus:

(4) $$\frac{u^{2}\left(\Delta R_{s}\right)}{R_{s}^{2}}=\frac{u^{2}\left(G_{s}\right)}{G_{s}^{2}}+\frac{Q_{s,1}^{-4}u^{2}\left(Q_{s,1}\right)+Q_{s,2}^{-4}u^{2}\left(Q_{s,2}\right)}{\left(Q_{s,1}^{-2}-Q_{s,2}^{-2}\right)}\;.$$

### 3.4. Method Applicability Study for Surface Roughness Evaluation

The most practically applied empirical model for $R(R_{q})$ is here reported [20]:

(5) $$R_{s}\left(R_{q}\right)=R_{0}\left(1+\frac{2}{\pi }\arctan\left(1.4{\left(R_{q}/\delta \right)}^{2}\right)\right)\;,$$

where $R_{0}$ is the surface resistance of a perfectly flat sample. Equation (5) shows that $R_{s}\to R_{0}$ when $R_{q}\ll \delta$, and $R_{s}\to 2R_{s,0}$ when $R_{q}\gg \delta$. In the case of pure copper, at 15 GHz and room temperature $\delta_{Cu}=$ 0.5 μm, hence $\Delta R_{s}\left(\Delta R_{q}\right)$ can be evaluated only with samples with $R_{q}∼\delta$ (i.e., 0.1 μm).

To determine the suitability of the designed resonator for this application, we compare the expected result from (5) with the minimum measurable ${\left(\Delta R_{s}\right)}_{min}$. ${\left(\Delta R_{s}\right)}_{min}$ depends on the sensitivity/uncertainty of the DR and on the sample $R_{s}$. In particular, the sensitivity function is $c=|\partial Q/\partial R_{s}|=Q^{2}/G$, thus ${\left(\Delta R_{s}\right)}_{min}={\left(\Delta Q\right)}_{min}G/Q^{2}$ with ${\left(\Delta Q\right)}_{min}=2Q\left(u\left(Q\right)/Q\right)$.

**Figure 4:**
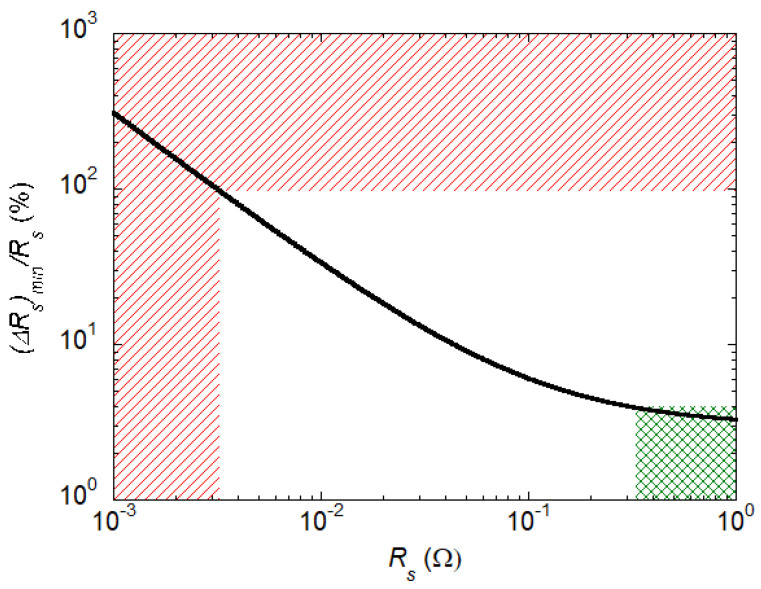
Percentage relative variation ${\left(\Delta R_{s}\right)}_{min}/R_{s}$ as a function of the surface resistance $R_{s}$ of the sample under investigation. The upper area where the DR is not sensitive to $R_{q}$ variation is marked with red oblique lines, while the bottom right green marked area is where ${\left(\Delta R_{s}\right)}_{min}/R_{s}<$ 4%.

With the use of (1) we simulate *Q* for sample $10^{-4}<R_{s}/\left(\Omega \right)<1$ with $l_{v}=4\times 10^{-5}$ [39], the brass rough base surface resistance $R_{base}=0.092\;\Omega$ and the same geometrical factor G=270 Ω for both base and sample. Then, ${\left(\Delta R_{s}\right)}_{min}/R_{s}$ is obtained and shown in Figure 4 setting u(Q)/Q=0.015. Since $\left(R_{s}\left(R_{q}\to \infty \right)-R_{s}\left(R_{q}\to 0\right)\right)/R_{s}\left(R_{q}\to 0\right)=1$, from Figure 4 one can assess that the minimum $R_{s}$ for which the determination of $R_{s}\left(R_{q}\right)$ is possible is $R_{s,min}=$ 3.2 mΩ. For lower $R_{s}$, the minimum distinguishable ${\left(\Delta R_{s}\right)}_{min}/R_{s}>1$, thus the $R_{q}$ effect on $R_{s}$ cannot be evaluated. However, since at 12.9 GHz for pure copper one has $R_{Cu}=$ 30 mΩ, the designed resonator can be reliably used with all good conductors. Thus, the resonator can detect effects on *R* corresponding to an evaluated minimum variation of $R_{q}=0.5\;\mu$m on Cu, based on Equation (5).

The different models in the literature rely on different shapes of the surface grooves (e.g., square or triangle [26]). Since the $R_{s}$ deviation between the different models is generally < 4% [26], one can ask if the designed DR would be able to determine this small difference, and then to ascertain the validity of one model over another. We highlight in green the plot area where ${\left(\Delta R_{s}\right)}_{min}/R_{s}<$ 4% in Figure 4. Thus, the dependence of the evaluation of $R_{q}$ on different models can be assessed only with highly resistive materials (e.g., stainless steel).

## 4. Conclusions

In this work, we explored the possibility of using a dielectric resonator (DR) for the study of the effects of surface roughness $R_{q}$ on the surface resistance *R* of good conductors. The high sensitivity of this kind of resonant measurement system is usually spoiled by large measurement uncertainties resulting from poor measurement repeatability. In order to improve the accuracy, we designed a new closed resonator with which the samples under investigation are set above the base of the resonator itself without the need of complete disassembly and re-mounting of the entire structure. We showed that the obtained measurement accuracy allows for the use of the designed DR for the study of the detrimental effects of $R_{q}$ on the surface resistance of conductors with R>3.2mΩ. Since at the working frequency and at room temperature the copper surface resistance is $R_{Cu}∼30\;m\Omega$, the theoretical achieved accuracy is sufficiently high to allow the use of this technique for the characterization of all standard good conductors. In particular, on copper the resolution on the average surface roughness evaluation was assessed to be ∼0.5 μm, which is comparable to the field penetration depth at ∼13 GHz. If this value is compared to those achievable with other techniques, which typically are <0.1 nm both for interferometric and mechanical profilometric methods [40], it is clear that the technique here proposed is sensitive on a different range of roughness scales. Of course, here the limiting factor is the loss of sensitivity of the surface resistance to the surface roughness of the material when this falls below the e.m. field penetration depth. For this reason, if the interest is in monitoring the surface roughness for the detrimental effects in microwave applications, then the extremely small resolution of interferometric methods is of little use since those roughness values do not affect the microwave performances (in terms of losses) of the material [15,16,20]. By contrast, for these applications the solution proposed here is sensitive enough with the great advantage of employing a simpler (and potentially more compact and cheaper) system than interferometers or atomic force microscopes.

## Data Availability

Not applicable.
